# Supplementary material for: Hyperoside and Quercitrin in Houttuynia cordata Extract Attenuate UVB-Induced Human Keratinocyte Cell Damage and Oxidative Stress via Modulation of MAPKs and Akt Signaling Pathway
Source: Antioxidants (Basel). 2022 Jan 24;11(2):221. doi: 10.3390/antiox11020221 (PMC8868276; doi:10.3390/antiox11020221)
Supplement: Supplementary file 1 [file antioxidants-11-00221-s001.zip › Figure S1.pdf]

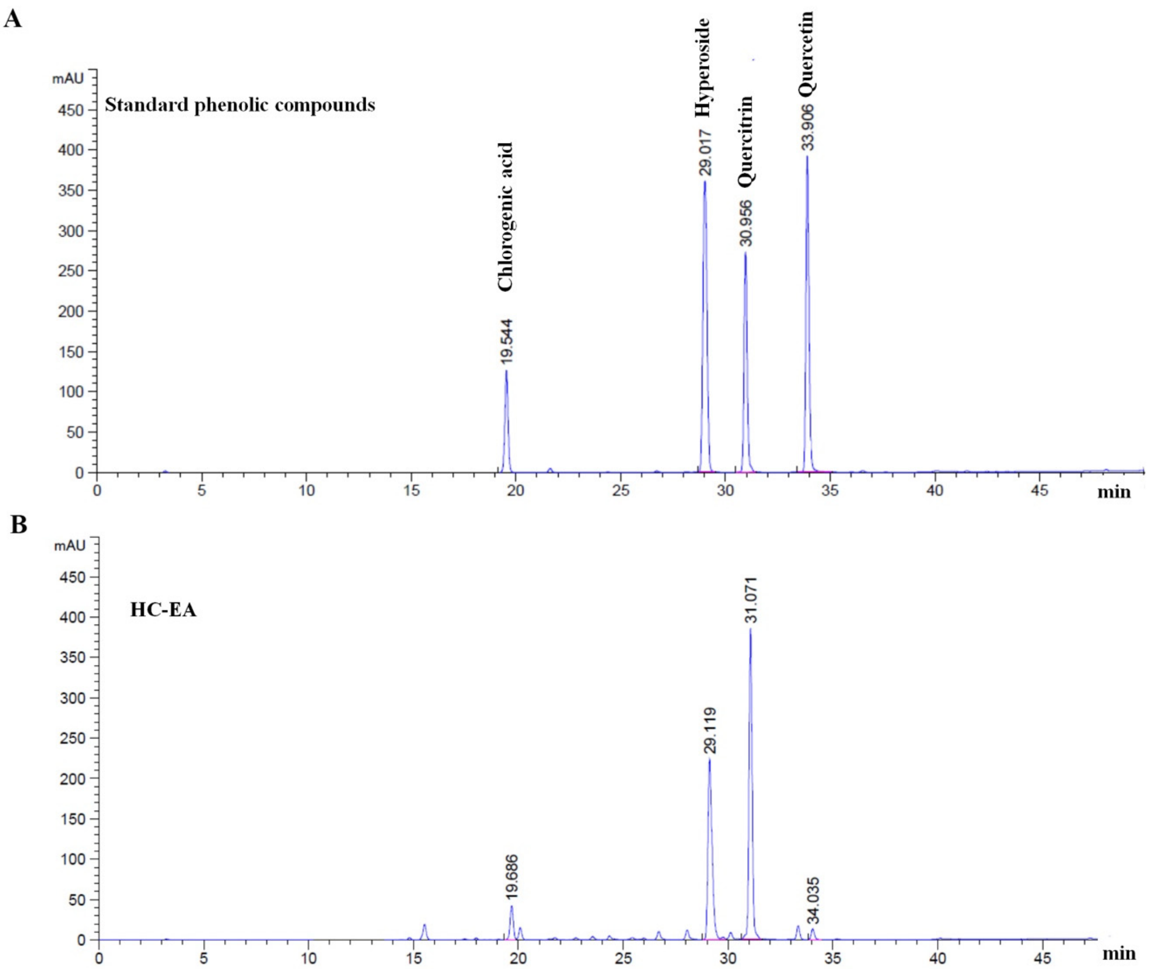

Figure S1
HPLC chromatogram of HC-EA and its standard compounds mixture. (A) HPLC chromatogram of the standard compounds of chlorogenic acid, hyperoside, quercitrin, and quercitrin. (B) HPLC chromatogram of HC-EA.
