# Supplementary material for: Hyperoside and Quercitrin in Houttuynia cordata Extract Attenuate UVB-Induced Human Keratinocyte Cell Damage and Oxidative Stress via Modulation of MAPKs and Akt Signaling Pathway
Source: Antioxidants (Basel). 2022 Jan 24;11(2):221. doi: 10.3390/antiox11020221 (PMC8868276; doi:10.3390/antiox11020221)
Supplement: Supplementary file 1 [file antioxidants-11-00221-s001.zip › Figure S2.pdf]

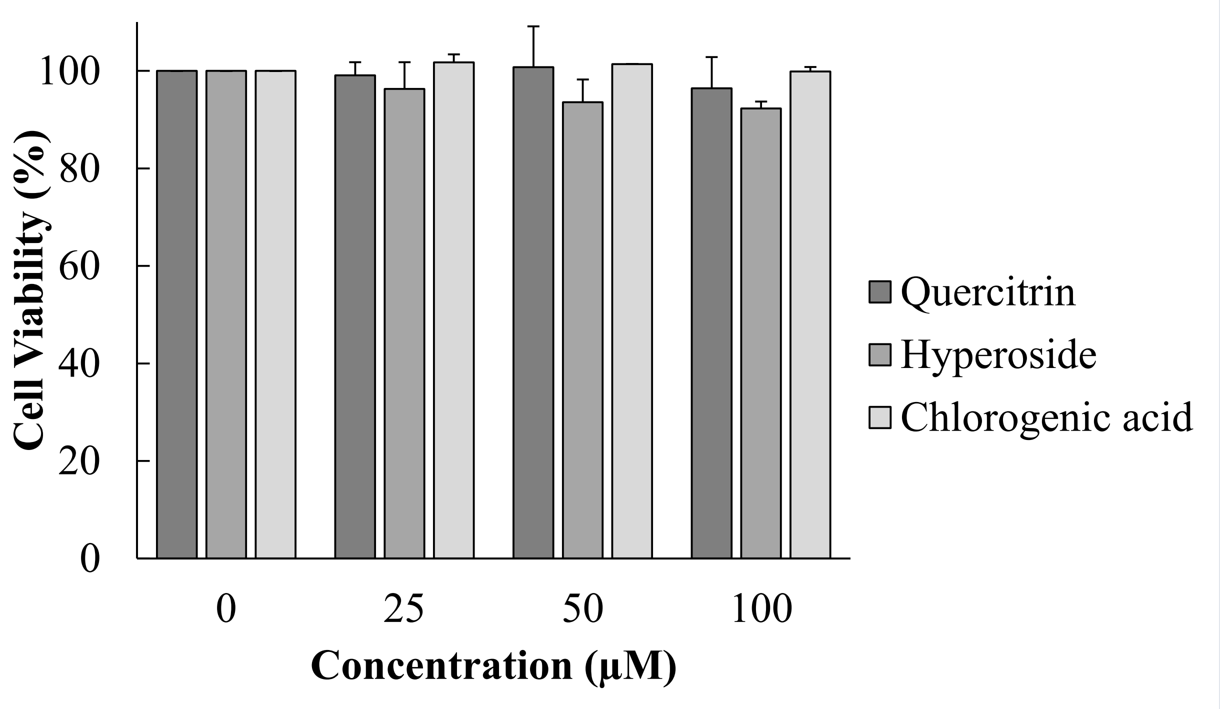

Figure S2
Effect of standard compounds on HaCaT cell viability. SRB assay was performed to investigate cell viabilities of the cells after treated with various concentrations of quercitrin, hyperoside, and chlorogenic for 48 hours.
